# Amisulpride augmentation of clozapine for treatment-refractory schizophrenia: a double-blind, placebo-controlled trial

**DOI:** 10.1177/2045125318762365

**Published:** 2018-03-08

**Authors:** Thomas R.E. Barnes, Verity Leeson, Carol Paton, Louise Marston, David P. Osborn, Raj Kumar, Patrick Keown, Rameez Zafar, Khalid Iqbal, Vineet Singh, Pavel Fridrich, Zachary Fitzgerald, Hemant Bagalkote, Peter M. Haddad, Mariwan Husni, Tim Amos

**Affiliations:** Centre for Psychiatry, Hammersmith Hospital Campus, Imperial College London, 7th Floor Commonwealth Building, Du Cane Road, London W12 0NN, UK; Centre for Psychiatry, Imperial College London, UK; Centre for Psychiatry, Imperial College London, UK; Oxleas NHS Foundation Trust, UK; Department of Primary Care and Population Health, University College London, UK; PRIMENT Clinical Trials Unit, University College London, UK; Division of Psychiatry, University College London, UK; Camden and Islington NHS Foundation Trust, London, UK; Tees, Esk and Wear Valley NHS Foundation Trust, Billingham, UK; Northumberland Tyne and Wear NHS Foundation Trust, Newcastle upon Tyne, UK; Newcastle University, Newcastle-upon-Tyne, UK; Lincolnshire Partnership NHS Foundation Trust, Lincoln, UK; Bradford District Care Trust, Bradford, UK; Derbyshire Healthcare NHS Foundation Trust, Derby, UK; North Essex Partnership University NHS Foundation Trust, Harlow, UK; Manchester Mental Health and Social Care NHS Trust, Manchester, UK; Nottinghamshire Healthcare NHS Foundation Trust, Nottingham, UK; Greater Manchester West Mental Health NHS Foundation Trust, Manchester, UK; University of Manchester, Manchester, UK; Central and North West London NHS Foundation Trust, London, UK; Northern Ontario School of Medicine, Ontario, Canada; Avon and Wiltshire Mental Health Partnership NHS Trust, Bristol, UK; School of Social and Community Medicine, University of Bristol, Bristol, UK

**Keywords:** amisulpride, antipsychotic medication, clozapine, clozapine augmentation, treatment-resistant schizophrenia

## Abstract

**Background:**

A second antipsychotic is commonly added to clozapine to treat refractory schizophrenia, notwithstanding the limited evidence to support such practice.

**Methods:**

The efficacy and adverse effects of this pharmacological strategy were examined in a double-blind, placebo-controlled, 12-week randomized trial of clozapine augmentation with amisulpride, involving 68 adults with treatment-resistant schizophrenia and persistent symptoms despite a predefined trial of clozapine.

**Results:**

There were no statistically significant differences between the amisulpride and placebo groups on the primary outcome measure (clinical response defined as a 20% reduction in total Positive and Negative Syndrome Scale score) or other mental state measures. However, the trial under recruited and was therefore underpowered to detect differences in the primary outcome, meaning that acceptance of the null hypothesis carries an increased risk of type II error. The findings suggested that amisulpride-treated participants were more likely to fulfil the clinical response criterion, odds ratio 1.17 (95% confidence interval 0.40–3.42) and have a greater reduction in negative symptoms, but these numerical differences were not statistically significant and only evident at 12 weeks. A significantly higher proportion of participants in the amisulpride group had at least one adverse event compared with the control group (*p* = 0.014), and these were more likely to be cardiac symptoms.

**Conclusions:**

Treatment for more than 6 weeks may be required for an adequate trial of clozapine augmentation with amisulpride. The greater side-effect burden associated with this treatment strategy highlights the need for safety and tolerability monitoring, including vigilance for indicators of cardiac abnormalities, when it is used in either a clinical or research setting.

## Introduction

In around a third of people with schizophrenia, the illness shows an unsatisfactory response to standard treatment with antipsychotic medication. Clozapine is the only antipsychotic medication with robust evidence for efficacy in strictly-defined treatment-resistant schizophrenia.^1,2^ But even then, an adequate response is seen in only 30–60% of patients prescribed this drug.^3,4^ To improve efficacy, clinicians commonly augment clozapine with another antipsychotic,^5^ although such a strategy has been found to have only modest benefit.^6–8^ The National Institute for Health and Care Excellence (NICE) guideline for the treatment of schizophrenia^9^ supports clozapine augmentation with a second antipsychotic when there has been an inadequate response to clozapine alone, noting that an adequate trial of such an augmentation might need to be up to 8–10 weeks, reflecting the findings of our own meta-analysis of relevant randomized controlled trials (RCTs).^10^

Clozapine is associated with potentially dangerous side effects such as agranulocytosis, myocarditis/cardiomyopathy and seizures as well as relatively common problems of potentially serious concern, such as weight gain, metabolic side effects and constipation. Thus, the criteria for selecting an augmenting antipsychotic drug might reasonably include a low liability to compound these side effects, as recommended by NICE. Given its perceived tolerability and safety advantages in relation to extrapyramidal side effects (EPS), weight gain and metabolic side effects, amisulpride may be considered particularly suitable for clozapine-augmentation therapy.^11^ This may be one reason why, in the UK, amisulpride is a relatively common choice to augment clozapine in clinical practice,^12^ despite the lack of robust clinical evidence on the potential risks and benefits of this drug combination. Another reason may be the perception that the selective dopamine D2/D3 blocking properties of amisulpride represent a complementary receptor profile to clozapine.^13^

The aims of this study were to further test the efficacy of an adequate trial of clozapine augmentation with amisulpride compared with placebo in treatment-resistant schizophrenia that had shown an insufficient response to clozapine and to assess the risks and possible adverse effects of such a trial. A report on this study to the funding body, the Health Technology Assessment programme of the National Institute for Health Research, has been published.^14^

## Methods

### Design and participants

The study was an individually randomized, double-blind, placebo-controlled, parallel-arm trial, approved by the London-Fulham Research Ethics Committee (Ref: 10/H0711/75), and the trial was registered (ISRCTN68824876). The participants were recruited from November 2011 to December 2014 from adult mental health services and all gave their written informed consent to take part in the study. The main inclusion criteria were treatment for at least 12 weeks at a stable dose of 400 mg or more of clozapine a day, unless the size of the dose was limited by side effects, a total score of 80 or greater at baseline on the Positive and Negative Syndrome Scale (PANSS),^15,16^ a Clinical Global Impression scale^17^ score of 4 or greater and a Social and Occupational Functioning Assessment Scale (SOFAS)^18^ score of 40 or less.

At baseline, up to three critical symptoms or behaviours that were refractory to treatment were identified for each participant. These phenomena had to have been persistent problems, judged clinically to have had a major adverse impact on a participant’s social function and community re-integration or been a major cause of psychological distress, or precluded discharge from hospital.

The total participation period was 12 weeks. Participants were randomized to 400 mg amisulpride a day or matching placebo capsules for the first 4 weeks, with the option of titrating up to 800 mg amisulpride or matching placebo capsules for the remaining 8 weeks. The amisulpride and placebo tablets had been encapsulated to look identical. A fully automated online randomization service was provided by the Clinical Trials Research Unit, University of Sheffield. The randomization sequence was generated using mixed blocks of two, four and six. Allocation was 1:1, stratified by recruitment site and high/low baseline PANSS score (low 80–92, high 93+). In addition, a 24 h unblinding service was provided by ESMS Global, Medical Toxicology Information Service Ltd, London.

#### Changes to methods after trial commencement

Additional sites were added as the trial progressed, taking the total number of study sites from 4 to 23. Prior to randomization of the first participant, electrocardiography was introduced to exclude cardiac contraindications to potentially high-dose antipsychotic medication, such as long QT syndromes, and establish a baseline reference for any subsequent cardiac monitoring. In line with a number of active, contemporaneous studies that were remunerating participants for their time, a payment to participants of £20 for each assessment was introduced, in recognition of any expenses incurred (e.g. travel) and inconvenience.

### Outcomes

The primary outcome measure was the proportion of patients with a criterion response threshold of a 20% reduction in total PANSS scale score. The inter-rater reliability of the PANSS ratings by researchers across the study sites was formally tested: the intraclass correlation for individual items was 0.63 (moderate agreement) and subscales at 0.86 (substantial agreement). The PANSS and the other rating scales were administered at baseline, 6 weeks and 12 weeks.

Negative symptoms were assessed using the PANSS negative symptom subscale. The impact on social and occupational function was measured using the SOFAS. The level of engagement with clinical services was assessed using the Service Engagement Scale (SES).^19^ Depressive symptoms were assessed using the Calgary Depression Rating Scale for Schizophrenia.^20^ Insight was assessed using the Schedule for the Assessment of Insight.^21^ The Antipsychotic Non-Neurological Side Effects Scale (ANNSERS),^22^ systematically and comprehensively assessed the full range of side effects, other than movement disorders, that are recognized as occurring with first- or second-generation antipsychotics. For this study, an enhanced version of the scale was generated (ANNSERS-E) by the addition of potential cardiac symptoms such as palpitations, dizziness and syncope. Additional side effects were assessed at baseline and at 12-week follow up, including body mass index, waist circumference, blood pressure and measurement of serum prolactin, plasma glucose and lipid profile (nonfasting sample). In line with best practice safety monitoring,^23^ an electrocardiogram (ECG) was carried out and reported on before the study medication was initiated.

With regard to EPS, drug-induced parkinsonism was assessed using the Simpson and Angus Extrapyramidal Side Effects Scale.^24,25^ The Barnes Akathisia Rating Scale^26^ was used to assess akathisia and the Abnormal Involuntary Movement Scale^17,27^ for rating tardive dyskinesia. The study researchers received thorough training on the use of these measures.

### Statistical analysis

Our sample size calculation was based on results from previous studies,^28,29^ which were comparable to the current study in terms of length of follow up, the nature of the intervention and the primary outcome, and a 20% or greater reduction in total PANSS score. To detect this criterion response in 30% of participants in the amisulpride arm and 10% in the placebo arm, with 90% power and an α of 0.05, would require 92 participants per group to complete the study (two sided).

All the main analyses were based on intention to treat. Baseline summary statistics by randomized group were calculated. Group differences in the primary outcome and other binary outcome measures were evaluated through the use of logistic regression after allowing for stratification by baseline symptom severity. Differences in continuous outcome measures were evaluated through corresponding analysis of covariance (ANCOVA) model, controlling for baseline symptom severity (the stratification variable), and baseline values of the outcome in question.

The 6-week data were used to determine whether there was benefit from the intervention earlier than the 12-week follow up. The 6-week outcome data were examined as a (tertiary) outcome, looking at the data longitudinally by applying mixed effects modelling using both 6 and 12-week outcomes and controlling for baseline values of the given measure. Data were analysed using Stata version 13 for Windows.^30^

## Results

**Figure 1.**
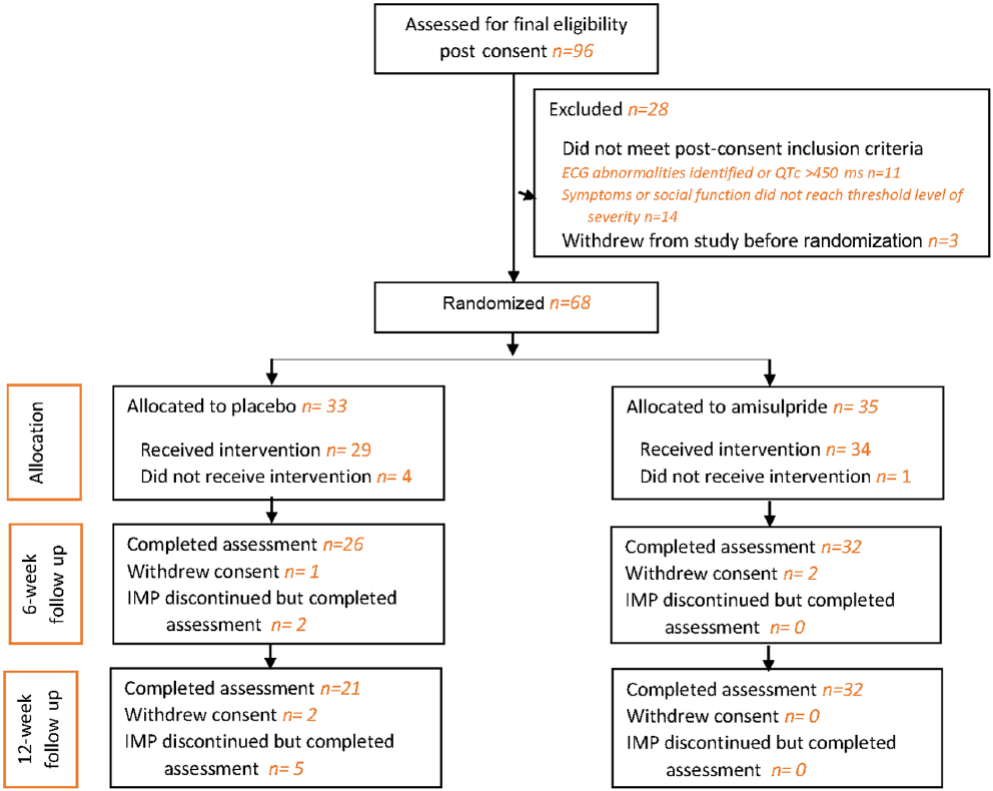
The CONSORT flow diagram. ECG, electrocardiogram; IMP, investigational medicinal product.

Of the 96 patients recruited, 68 were randomized, with 52 completing their assigned treatment regimen and assessment at the 12-week follow-up. Figure 1 is the CONSORT diagram of progress through the phases of the trial. Table 1 shows the demographic characteristics and status of the participants in the two treatment groups at baseline while Table 2 provides information on the clinical characteristics. The critical symptoms or behaviours refractory to treatment that characterized the study sample were identified by the responsible clinical teams. Positive symptoms were the most common: hallucinations were reported for 51% of participants, delusions for 43%, and suspiciousness/persecutory or paranoid ideas for 33%. Reduced social interaction was identified as a problem for 37%. Anxiety was relatively common, being identified as a persistent issue for 35% of participants, while depression was a key symptom in only 9%. General negative symptoms were mentioned for 12% of participants but, more specifically, 20% of participants were reported as exhibiting a lack of drive, motivation, volition or spontaneity.

**Table 1.** Demographic characteristics and status of participants at baseline, by randomized groups.

Variable	Amisulpride		Placebo	
	*n*/*N* or mean	% or (SD)	*n/N* or mean	% or (SD)
Male	24/35	69	23/33	70
Age: years	39	(11)	40	(10)
Ethnicity: white	28/35	80	24/33	73
Living alone	12/32	38	11/28	39
Living with parents	5/32	16	8/28	29
Living with others	15/32	47	9/28	32
Owner occupied flat or house	0/34	0	0/29	0
Flat or house rented	19/34	56	21/29	72
Other accommodation	15/34	44	8/29	28
Not in paid employment because of treatment	24/25	96	23/25	92
Currently an inpatient	5/35	14	4/33	12
Psychiatric inpatient in the last 3 months	1/22	5	0/20	0

SD, standard deviation.

**Table 2.** Clinical characteristics of participants at baseline, by randomized groups.

Variable	Amisulpride		Placebo	
	*n/N* or mean	% or (SD)	*n/N* or mean	% or (SD)
*Primary psychiatric diagnosis*				
Schizophrenia	32/34	94	29/30	97
Schizophreniform disorder	1/34	3	0/30	0
Schizoaffective disorder	1/34	3	0/30	0
Psychosis NOS	0/34	0	1/30	3
*Medication*				
Any antidepressant	15/35	43	13/33	39
Any antipsychotic (excluding clozapine and amisulpride)	3/35	9	1/33	3
Any mood stabilizer: lithium, valproate, carbamazepine or lamotrigine	9/35	26	4/33	12
*Clinical assessment*				
Mental state: PANSS	93	(13)	98	(24)
PANSS high score (stratification variable)	16/35	46	14/33	42
PANSS negative symptom subscale score	25	(6)	25	(7)
Depression: CDSS median (IQR)	5	(1–10)	5	(2–8)
Social function: SOFAS median (IQR)	35	(32–39)	35	(30–40)
Service engagement: SES median (IQR)	8	(4–13)	10	(4–18)
Insight: SAI median (IQR)	12	(8–13)	12	(9–14)
*Side effects*				
ANNSERS-E median (IQR)	16	(11–22)	13	(10–24)
BARS: median (IQR)	0	(0–2)	2	(0–2)
Akathisia present (global item score ⩾2)	3/33	9	4/31	13
AIMS positive: tardive dyskinesia	4/35	11	4/33	12
EPSE: median (IQR)	0.1	(0–0.3)	0.1	(0–0.3)
Parkinsonism present (total score ⩾3)	10/29	34	6/24	25

AIMS, Abnormal Involuntary Movement Scale; ANNSERS-E, Antipsychotic Non-Neurological Side Effects Scale enhanced version; BARS, Barnes Akathisia Rating Scale; CDSS, Calgary Depression Rating Scale for Schizophrenia; EPSE, Extrapyramidal Side Effects Scale; IQR, interquartile range; NOS, Not Otherwise Specified; PANSS, Positive and Negative Syndrome Scale; SAI, Schedule for the Assessment of Insight; SD, standard deviation; SES, Service Engagement Scale; SOFAS, Social and Occupational Functioning Assessment Scale.

At the 6-week study assessment, the mean PANSS total score was higher for the placebo group [85 (standard deviation (SD) 23] compared with the amisulpride group [80 (SD 15)], although the same proportion (25%) had a 20% drop in PANSS score from baseline in both groups. Median SES score was lower in the placebo group [7 interquartile range (IQR) 4–14] compared with the amisulpride group [10 (IQR) 4–13]. All other standardized scales showed similar scores between the treatment groups.

A 20% or greater reduction in PANSS total score by 12 weeks was found in 44% of those participants in the amisulpride group compared with 40% of those assigned to placebo. As can be seen from the data presented in Table 3, this reflects numerically higher odds, odds ratio (OR) 1.17 [95% confidence interval (CI) 0.40–3.42] for the amisulpride group for achieving this criterion level of reduction in PANSS total score.

**Table 3.** Outcomes in terms of the amisulpride intervention.

Variable	OR or coefficient	95% CI
**Primary outcome**		
>20% reduction in PANSS from baseline (OR)	1.17	(0.40, 3.42)
**Secondary outcomes**		
PANSS negative symptom subscale	−0.71	(−3.22, 1.81)
Service engagement: SES	1.17	(−1.63, 3.97)
Depression: CDSS	0.23	(−1.54, 2.00)
Insight: SAI	0.02	(−1.33, 1.37)
**Side effects**		
*Non neurological*		
ANNSERS-E	1.58	(−3.60, 6.76)
*Metabolic/endocrine side effects*		
Weight	0.79	(−1.40, 2.99)
Body mass index	−0.02	(−1.05, 1.01)
Waist circumference	1.05	(−2.33, 4.42)
Systolic blood pressure (mmHg)	3.49	(−3.66, 10.63)
Diastolic blood pressure (mmHg)	3.33	(−1.65, 8.31)
Serum prolactin (ng/ml)	50.47	(−8.86, 109.80)
Ln* serum prolactin	1.43	(0.71, 2.14)
Plasma glucose (mmol/liter): nonfasting blood sample	0.66	(−0.22, 1.54)
Total cholesterol (mmol/liter)	0.48	(−0.11, 1.07)
HDL cholesterol (mmol/liter)	0.09	(−0.23, 0.41)
LDL cholesterol (mmol/liter)	0.11	(−0.62, 0.85)
Triglycerides (mmol/liter)	0.78	(−0.10, 1.65)
*Motor side effects*		
BARS: akathisia present^$^ (global item score ⩾2) (OR)	0.35	(0.06, 2.09)
Tardive dyskinesia: AIMS positive^$^ (OR)	0.37	(0.03, 4.34)
EPSE	−0.04	(−0.22, 0.14)
Parkinsonism present (total score ⩾3)^$^ (OR)	0.63	(0.18, 2.20)

* Logarithmic transformation.

$ Unadjusted result, too few events to do an adjusted analysis.

AIMS, Abnormal Involuntary Movement Scale; ANNSERS-E, Antipsychotic Non-Neurological Side Effects Scale enhanced version; BARS, Barnes Akathisia Rating Scale; CDSS, Calgary Depression Rating Scale for Schizophrenia; CI, confidence interval; EPSE, Extrapyramidal Side Effects Scale; HDL, high-density lipoprotein; LDL, low-density lipoprotein; OR, odds ratio, all other results are coefficients; PANSS, Positive and Negative Syndrome Scale; SAI, Schedule for the Assessment of Insight; SES, Service Engagement Scale.

Table 4 presents the results of mixed effects modelling to take time into account in terms of the amisulpride intervention. There were no differences between the amisulpride and placebo groups. However, for the study population as a whole, these analyses reveal a time effect associated with more than 20% reduction in PANSS; the odds of a reduction in PANSS of more than 20% at 12 weeks is 4.19 times that of 6 weeks (95% CI 1.20–14.56), controlling for baseline PANSS score and including the randomized condition. Likewise, PANSS negative subscale scores show a slight decrease in score at 12 weeks compared with 6 weeks (–1.32; 95% CI –2.20, –0.44) controlling for baseline negative PANSS score and including the randomized condition.

**Table 4.** Mixed effects modelling to take time (baseline, 6 weeks and 12 weeks) into account in terms of the amisulpride intervention.

Variable	OR or coefficient	95% CI
*Primary outcome*		
20% reduction in PANSS from baseline (OR)	1.43	(0.24, 8.44)
*Secondary outcomes*		
PANSS negative symptom subscale	−0.60	(−2.58, 1.39)
Service engagement: SES	1.75	(−0.54, 4.04)
Depression: CDSS	0.19	(−1.10, 1.49)
Insight: SAI	−0.52	(−2.32, 1.28)
*Side effects*		
ANNSERS-E	3.11	(−0.91, 7.13)
BARS: akathisia present (global item score ⩾2) (OR)	0.29	(0.01, 7.82)
Tardive dyskinesia: AIMS positive (OR)	0.18	(0.00, 32.67)
Parkinsonism: EPSE	0.05	(−0.09, 0.19)

AIMS, Abnormal Involuntary Movement Scale; ANNSERS-E, Antipsychotic Non-Neurological Side Effects Scale enhanced version; BARS, Barnes Akathisia Rating Scale; CDSS, Calgary Depression Rating Scale for Schizophrenia; CI, confidence interval; EPSE, Extrapyramidal Side Effects Scale; OR, odds ratio, all other results are coefficients; PANSS, Positive and Negative Syndrome Scale; SAI, Schedule for the Assessment of Insight; SES, Service Engagement Scale.

### Side effects

An early check on cardiac side effects, at 7–10 days after starting study medication, discovered a greater frequency in the amisulpride group: five participants reported shortness of breath, five reported dizziness and one reported irregular heartbeat. In the placebo group, one participant reported dizziness.

The data in Table 3 regarding side-effect assessment using the ANNSERS-E reveal that, at 12 weeks, the mean ANNSERS-E total score in those participants assigned to amisulpride was 1.58 (95% CI –3.60, 6.76) points higher on average than in the placebo group.

By 12 weeks, mean weight, waist circumference and blood pressure were greater in the amisulpride group than in the placebo group (see Table 3). Serum prolactin concentration was higher in the amisulpride group than in the placebo group by, on average, 50.5 ng/ml (95% CI –8.86, 109.80), as was mean plasma glucose concentration, which was on average 0.66 mmol/liter (95% CI –0.22, 1.54) higher.

During the course of the study, 65 adverse events were reported for 31 participants; more of these events were in the amisulpride intervention group than the placebo group (47 *versus* 18). Most of the adverse events reported were characterized as mild and eventually resolved. Almost a third of adverse events in the amisulpride group were judged by the reporting clinician to be either ‘probably’ or ‘definitely’ related to the study medication compared with a little over a tenth in the placebo group. In the amisulpride group, 60% (*n* = 21/35) had at least one adverse event compared with 30% (*n* = 10/33) in the control group (*p* = 0.014). Forty percent of the adverse events in the amisulpride group were cardiac symptoms (compared with 11% of the adverse events in the placebo group): dizziness and breathlessness were the most common, each reported by six participants, with postural dizziness, irregular heartbeat and tachycardia each reported by two participants. However, serious adverse events were rare and none related to the study medication, with one participant experiencing such an event in the amisulpride group and two participants in the placebo group.

## Discussion

### Efficacy

The only other double-blind, placebo-controlled study testing amisulpride augmentation of clozapine in patients with schizophrenia that has shown an insufficient response to clozapine treatment was by Assion and colleagues.^31^ These investigators concluded that this was a potentially helpful treatment option in such cases but acknowledged the limitations of their small sample size (*n* = 16) and relatively short, 6-week follow up. Our trial had a much larger sample size and longer follow up but we under recruited against our target sample size and therefore the power of any statistical analysis to detect significant differences between the active and placebo groups was limited. We found no statistically significant differences between the amisulpride and placebo groups on mental state measures.

Participants in the amisulpride group had modestly higher odds of being clinical responders by the end of the 12-week study period, the response criterion being a 20% or greater reduction in the total PANSS score. This advantage was not evident at 6 weeks, possibly reinforcing earlier indications that an adequate trial of clozapine augmentation with a second antipsychotic may be at least 10–12 weeks,^10,32^ that is, longer than the 4–6 weeks usually considered adequate for the treatment of an acute psychotic episode. However, this numerical difference in the proportion of responders was not significant. As we did not achieve the target number of participants required to power the study, we cannot be certain whether the lack of significant difference between the groups is a type II error or reflects a true lack of efficacy for this augmentation strategy. Further, the emergence of a difference in treatment response between the groups by the 12-week assessment that had not been present at 6 weeks may have implications for the duration of an adequate trial of this drug combination. Whether in a clinical or research setting, treatment for more than 6 weeks may be required for any potential benefit to emerge. Another, nonsignificant finding was a greater reduction in the PANSS negative symptom subscale score by 12 weeks in those participants assigned to amisulpride, compared with the placebo group. This seems to be in accord with earlier reports of a greater improvement in negative symptoms than positive symptoms in randomized studies where clozapine augmentation with a second antipsychotic for treatment-refractory schizophrenia has proved to be beneficial^28,33^ as well as some limited evidence for improvement in negative symptoms with amisulpride monotherapy.^34–37^

When considering the findings, it should be borne in mind that a response criterion of a 20% or greater reduction in total PANSS score for people with treatment-refractory schizophrenia may be of limited clinical relevance. Its interpretation requires an understanding of the meaning of scores on a scale rarely used in clinical practice. Further, as Leucht and colleagues^38^ demonstrated, even a 25% reduction in the PANSS total score may only reflect a reduction of the Clinical Global Impression scale score by one severity step. Given the marked heterogeneity of the clinical presentation of treatment-refractory schizophrenia, a more clinically relevant outcome measure in future studies of this kind might be an individualized response criterion, based on the change in severity of each participant’s critical target symptoms. This last point is reinforced by the diverse clinical profiles presenting in this study sample. While persistent positive symptoms were the most common features at baseline judged to be of clinical significance by the mental health professionals providing care, some participants presented other such target symptoms and behaviours, including anxiety, reduced social interaction, and negative symptoms in the avolition/amotivation domain.

### Side effects

Amisulpride was chosen for this study because of the robust evidence for safety and tolerability benefits, particularly a low risk of compounding characteristic clozapine side effects. While amisulpride is recognized as an antipsychotic drug with a relatively high risk of causing hyperprolactinaemia,^39^ it causes little or no weight gain and has a relatively low liability for diabetes, lipid abnormalities and EPS.^40,41^ With regard to cardiac side effects, QT interval prolongation and the potentially fatal arrhythmia, torsade de pointes, are not uncommon with overdose^42^ but the risk at therapeutic dosages is rather uncertain.^43,44^

Using the ANNSERS-E scale, we found a greater side-effect burden in those participants assigned to the clozapine–amisulpride combination. Their mean ANNSERS-E total over the course of the study was slightly higher than the equivalent score in the placebo group. However, our separate scale assessments of EPS, such as akathisia and parkinsonism, revealed that these were not likely to be treatment-emergent problems with amisulpride augmentation of clozapine, despite the ‘high rates’ of tremor, bradykinesia and akathisia previously reported with the combination.^31,45^

Considering the adverse events reported during the course of the study, 60% of participants in the amisulpride group had reported at least one, compared with a respective figure of 30% for the placebo group. Cardiac symptoms proved to be a relatively common prompt for an adverse event report, occurring much more commonly in the amisulpride group. Further, an additional check for any emerging cardiac symptoms in the 7–10 days after starting study medication revealed that problems such as shortness of breath or dizziness were more common in the amisulpride group. Amisulpride augmentation was also associated with raised serum prolactin, an expected side effect that also provides some indirect but reassuring evidence of adherence to the study medication.

### Mechanism of action

One proposed criterion for the choice of an augmenting antipsychotic in patients on clozapine is a complementary receptor profile, essentially potent D2 dopamine receptor blockade.^13,46,47^ This was partly the rationale for choosing amisulpride for this study: it preferentially binds to dopamine D2 and D3 receptors in limbic rather than striatal brain structures^48,49^ and has low affinity for other dopamine receptor subtypes, although it also has affinity for a range of other receptors, including serotonergic, histaminergic and adrenergic receptors.

However, the limited benefit seen with amisulpride in this study suggests that the notion that potent D2 blockade is a key determinant of response when adding a second antipsychotic to treat clozapine-unresponsive illness may be simplistic. Treatment-refractory schizophrenia may have a more complex pathophysiology than illness showing a good therapeutic response to standard antipsychotic therapy; the underlying pathophysiology may even be nondopaminergic.^50–52^ For example, dopamine synthesis capacity is lower in those patients with a treatment-resistant illness (indeed, no different from healthy controls) than in those with a responsive illness.^53^ It has been speculated that treatment-resistant illness may benefit from a multisite receptor effect rather than a stronger antidopaminergic effect.^6,54^

## Conclusion

We found no significant differences in therapeutic efficacy between the amisulpride-augmented group and the placebo-augmented group. This is consistent with the results of meta-analyses of studies of clozapine augmentation strategies, which have either been negative or identified only small positive findings based on single or outlying studies.^7^ In our study, amisulpride augmentation was associated with a slightly greater chance of improvement to a criterion level of overall symptom reduction within 12 weeks and a modest improvement in negative symptoms, but these observed differences failed to reach statistical significance. Further, despite amisulpride being chosen for its favourable tolerability and safety profile, when combined with clozapine treatment in this study it was associated with a greater side-effect burden, including cardiac symptoms. The identification of such problems may partly reflect the thorough assessment of side effects in this study, which was more systematic and comprehensive than is generally conducted in clinical trials of antipsychotics.^55^ These findings have implications for the nature and frequency of safety and tolerability monitoring of clozapine augmentation with amisulpride and, potentially, with other antipsychotic medications, in both clinical and research settings.

The lack of significant benefit with amisulpride seen in this trial challenges the rationale of potent dopamine D2 receptor blockade as a key criterion for selecting an augmenting antipsychotic to treat clozapine-unresponsive illness. Nevertheless, as the trial was underpowered, this treatment strategy is still worthy of further investigation in larger studies. Future trials of such a treatment strategy should have a sample size that provides adequate statistical power and be of sufficient duration, taking into account that a clinical response may not be evident within the 4–6-week follow-up period usually considered adequate in studies of antipsychotic treatment of acute psychotic episodes. Whether such trials are feasible remains uncertain, given the continuing challenge of recruitment in mental health studies in the NHS.^56,57^
